# Diagnosing multiple system atrophy: current clinical guidance and emerging molecular biomarkers

**DOI:** 10.3389/fneur.2023.1210220

**Published:** 2023-09-29

**Authors:** Meghana Goolla, William P. Cheshire, Owen A. Ross, Naveen Kondru

**Affiliations:** ^1^Department of Neuroscience, Mayo Clinic, Jacksonville, FL, United States; ^2^Department of Surgery, University of Illinois, Chicago, IL, United States; ^3^Department of Neurology, Mayo Clinic, Jacksonville, FL, United States; ^4^Department of Clinical Genomics, Mayo Clinic, Jacksonville, FL, United States; ^5^Department of Biology, University of North Florida, Jacksonville, FL, United States

**Keywords:** multiple system atrophy (MSA), autonomic dysfunction, α-synuclein, biomarkers, protein amplification assays, RT-QuIC

## Abstract

Multiple system atrophy (MSA) is a rare and progressive neurodegenerative disorder characterized by motor and autonomic dysfunction. Accurate and early diagnosis of MSA is challenging due to its clinical similarity with other neurodegenerative disorders, such as Parkinson’s disease and atypical parkinsonian disorders. Currently, MSA diagnosis is based on clinical criteria drawing from the patient’s symptoms, lack of response to levodopa therapy, neuroimaging studies, and exclusion of other diseases. However, these methods have limitations in sensitivity and specificity. Recent advances in molecular biomarker research, such as α-synuclein protein amplification assays (RT-QuIC) and other biomarkers in cerebrospinal fluid and blood, have shown promise in improving the diagnosis of MSA. Additionally, these biomarkers could also serve as targets for developing disease-modifying therapies and monitoring treatment response. In this review, we provide an overview of the clinical syndrome of MSA and discuss the current diagnostic criteria, limitations of current diagnostic methods, and emerging molecular biomarkers that offer hope for improving the accuracy and early detection of MSA.

## Introduction

Multiple system atrophy (MSA) is a rare, sporadic, progressive neurodegenerative disorder that manifests with variable combinations of parkinsonism, cerebellar ataxia, and autonomic failure. MSA is very difficult to diagnose in its early stages. As its neurologic deficits impact multiple organ systems, MSA patients may initially be seen by non-neurologic specialists such as gastroenterologists or urologists years before their condition is recognized as a neurologic disorder. During the early stages of the disease process, patients are often misdiagnosed, and even after eventual referral to a neurologist, the diagnosis may be further prolonged by the clinical resemblance of MSA to other Parkinsonian or cerebellar disorders. Once clinically suspected or diagnosed, MSA progresses rapidly with most patients requiring a wheelchair or bedridden within 3–5 years. The prognosis is poor with limited treatment options and no cure (1, 2).

**Figure 1:**
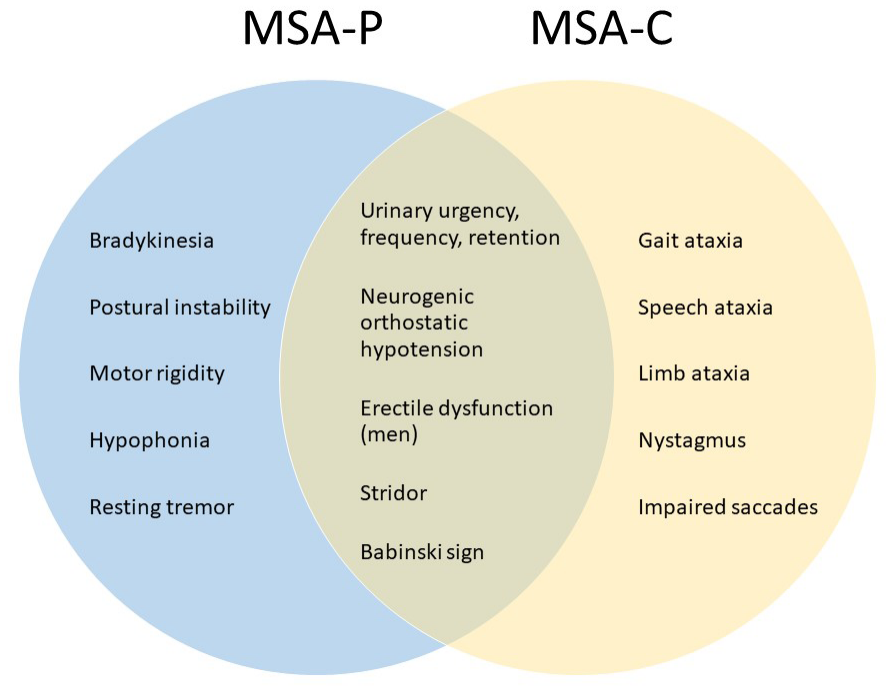
Clinical signs for diagnostic assessment of MSA subtypes.

MSA is classified into two major clinical subtypes: MSA-P (with predominantly parkinsonian deficits) and MSA-C (with predominantly cerebellar deficits). Figure 1 summarizes some common clinical observations (3, 4). The MSA-P subtype is characterized pathologically by striatonigral degeneration and presents clinically with motor traits aligned with Parkinson’s disease (PD), such as bradykinesia, postural instability, muscle rigidity, hypophonia, and resting tremor, but unlike PD, the response to levodopa is poor. The MSA-C subtype is characterized pathologically by cerebellar and pontine atrophy (3, 4) and presents clinically with impaired gait, eye, speech, and limb coordination. MSA-C resembles some of the spinocerebellar ataxias (SCA) but is distinguished by the presence of autonomic failure (5). In some patients, MSA is manifested with mixed parkinsonism and cerebellar deficits and is typically classified according to which clinical signs occurred first or are the most severe. The pathologic hallmarks are glial cytoplasmic inclusions (GCI) consisting of misfolded α-synuclein protein in oligodendroglia (1, 6, 7), and neuronal loss occurring in striatonigral and olivopontocerebellar systems (7, 8). A relatively new addition to the MSA spectrum is minimal change MSA (MC-MSA) (9, 10). MC-MSA is also characterized by neuronal loss primarily restricted to the substantia nigra and locus coeruleus Its relationship to other forms of MSA is still under investigation, but it is thought to represent an early stage of MSA, or a distinct variant caused by different genetic or environmental factors. Further research is needed to define clinical and distinct features of MC-MSA within the broader MSA spectrum (10).

The criteria to diagnose MSA distinguish 4 subcategories of clinical certainty: neuropathologically established MSA, clinically established MSA, clinically probable MSA, and possible prodromal MSA (7). A definitive diagnosis of MSA requires neuropathological evidence of α-synuclein within GCI and neurodegenerative changes in the striatonigral or olivopontocerebellar regions (7, 9, 11). The highest concentration of GCI is contained within the basal ganglia (9). The degree of the inclusion concentration correlates with the severity and progression of MSA, showing that the glial inclusions are likely involved in the pathogenesis (9).

The clinical diagnosis of MSA remains challenging even among experts since its presenting features overlap with other disorders such as PD, Lewy body disease (LBD), SCA, or progressive supranuclear palsy (PSP) (12). One study found that the current accuracy of the clinical diagnosis through neuropathologic testing is assumed to be 62% after autopsy confirmation (13); Other studies have also indicate that the clinical diagnosis of multiple system atrophy (MSA) is often inaccurate, even among experienced neurologists (14, 15). For instance, in a autopsy-confirmed MSA study, the sensitivity of clinical diagnosis at the first visit was only 56%, and the positive predictive value was 76%. However, the last visit, the sensitivity had improved to 69%, and the positive predictive value had increased to 80% indicating diagnostic accuracy may improve with the disease progression (14). However, misdiagnosis can lead to delays in treatment, which can have a negative impact on the patient’s quality of life.

In a study conducted by Joutsa et al., it was found that general neurologists exhibit a relatively low diagnostic accuracy for parkinsonism syndromes, with approximately 25% of diagnoses being incorrect (16). However, when compared to multiple system atrophy (MSA), the diagnostic accuracy for parkinsonism syndromes as a whole is slightly higher. The challenge in accurately diagnosing MSA lies in the overlap of clinical features between MSA and other disorders such as PD and PSP. Although consensus clinical criteria to diagnose MSA have improved (7), the need for a specific biological biomarker is still unmet (7, 17). The availability of a noninvasive diagnostic molecular biomarker would facilitate early diagnosis and greatly improve the management of disease as well as to advance treatments (3, 7, 17).

The remaining three diagnostic categories are based on clinical evaluation and disease progression. The clinical subset has definitive criteria to be met, such as age greater than 30 years, a negative family history, and disease progression in line with MSA (3, 7). The clinically established category requires a magnetic resonance imaging (MRI) study showing evidence of neurodegeneration patterns consistent with MSA, while the clinically probable category does not. Other clinical indicators for the diagnosis of MSA include urogenital dysfunction (urinary retention or incontinence) and other autonomic dysfunction, especially neurogenic orthostatic hypotension (18). Another criterion used to distinguish MSA is the decreased or ineffective response of parkinsonism to levodopa therapy (9, 19). Nevertheless, as some MSA patients will initially respond to levodopa, this criterion cannot be solely used to differentiate MSA from PD (20). The category of possible prodromal MSA was added to ensure that MSA is considered in the differential diagnosis early in the disease’s progression. The intent is to capture patients who show initial signs of extrapyramidal motor or autonomic dysfunction but do not yet show definitive signs of MSA (4, 21, 22). Minimal change MSA is an early pathologic form with minimal neuronal loss but with glial cytoplasmic inclusions (23). Additionally, it is essential for healthcare providers to consider the impact of the diagnosis on the patient and their family, as MSA is a debilitating disease with a poor prognosis. This is particularly challenging for clinicians that provide explicit documentation of MSA early in the course of an uncertain disease. For more information on the current diagnostic criteria and guidelines for MSA diagnosis, the reader is referred to the cited references (7, 9). Therefore, further research is needed to develop reliable and specific biomarkers for MSA diagnosis, which will facilitate early detection and intervention.

## Current imaging approaches to aid clinical diagnosis

In addition to clinical signs, Magnetic resonance imaging (MRI) and positron emission tomography (PET) are the crucial imaging tools employed in MSA diagnosis. These imaging modalities provide valuable insights into the underlying neurodegenerative processes and help differentiate MSA from other parkinsonian disorders, including Parkinson’s disease (PD) and atypical parkinsonism.

MRI findings in MSA include significant atrophy in specific brain regions such as putamen, middle cerebellar peduncle, pons, and cerebellum (7, 24). A sentinel finding on MRI is the presence of a “hot-cross bun” pattern on T2-weighted and FLAIR images, characterized by hyperintensity in the pontine tegmentum, usually accompanied by cerebellar atrophy. While this finding is not specific to MSA, it provides evidence to support the diagnosis when clinical features are taken into consideration (3, 25). It is worth mentioning that the “hot-cross bun” sign can also be observed in other neurodegenerative disorders, such as PSP. Furthermore, specific alterations within the putamen, such as atrophy of the putamen and increased diffusivity in the putamen and middle cerebellar peduncle (MCP) help to establish a diagnosis of MSA (7, 26). The atrophy of the putamen is typically observed in MSA patients and can aid in differentiating MSA from other parkinsonian disorders. On the other hand, altered diffusivity within the putamen can provide additional supportive evidence for MSA diagnosis (26). While MRI can be a valuable tool for diagnosis of neurodegenerative disorders, in clinical practice, imaging in the early stages of PD is usually normal (26, 27). The signatures, such as the ‘hot-cross bun’ discussed above, are more apparent as the disease process progresses, making MRI a tool to distinguish between disorders with greater progression of disease (27).

Whereas MRI scans of PD and MSA are indiscernible in early disease course, fluorodeoxyglucose 18F-FDG-PET imaging has emerged as a valuable technique for differentiating MSA from PD and other parkinsonian disorders (3, 28). 18F-FDG-PET utilizes a special tracer glucose to evaluate the uptake within tissues, PET scanning allows for different tracers to be utilized for the different structures and conditions being evaluated (27). 18F-FDG-PET signatures provide valuable insights into the metabolic patterns specific to each condition. Putaminal and cerebellar hypometabolism are characteristic 18F-FDG-PET findings in MSA, serving as supportive criteria in the diagnosis (11). These metabolic patterns in MSA are distinct from the hypometabolism observed in the parietal region in PD patients. Thus, 18F-FDG-PET imaging can contribute significantly to the accurate diagnosis of MSA, especially when combined with other clinical and imaging findings. The signature sign with MSA patients was a reduced tracer uptake within the basal ganglia and cerebellum, while in PD patients it was hypometabolism in the parietal region (29).

In the differential diagnosis of PD vs. MSA, evidence of cardiac noradrenergic denervation using ^123^I-metaiodobenzylguanidine (^123^I-MIBG-) single photon emission computed tomographic (SPECT) imaging has proven useful in identifying PD and excluding MSA (29).

It is important to note that while the use of MIBG imaging, specifically 123I-MIBG-SPECT can help distinguish between PD and MSA in advanced stages of the diseases, its reliability in the early stages is limited. Most MSA patients exhibit normal sympathetic innervation of the myocardium; however, mild reductions in cardiac sympathetic innervation have been reported in some cases (30). It is crucial to exercise caution when interpreting 123I-MIBG-SPECT results in the early differential diagnosis between PD and MSA, as patients with early PD may express non-pathologic cardiac sympathetic innervation (29). Efforts to develop a biomarker of radiolabeled antibodies or compounds utilized through PET scanning to specifically detect α-synuclein are under investigation. Current laboratory studies using rodent models show some promise in detecting extracellular α-synuclein deposits. However, further testing is required for the detection of intracellular aggregation of the α-synuclein present in MSA via PET (31, 32).

Dopamine transporter SPECT (DAT-SPECT) is another testing module which aids in diagnosing MSA (33). This imaging modality detects the degeneration of the nigrostriatal pathway linked to dopaminergic pathophysiologies, such as PD and MSA (34). DAT-SPECT utilizes radiotracers specific for dopamine transporters in the presynaptic terminals to assess the activity of dopamine activity and density (35). Studies show that there are differences in DAT-SPECT binding to help differentiate PD and MSA-P, with lower and more symmetrical binding in MSA-P than PD (33, 36). While there are some attempts to differentiate between different nigrostriatal and dopaminergic pathophysiologies, the findings are not specific enough limiting DAT-SPECT in its utility to make differential diagnosis (34). Due to these limitations, dopaminergic imaging is not reliable in differentiating between the causes of parkinsonism reliably and is not recommended for clinical practice (34).

In conclusion, the utilization of MRI, 18F-FDG-PET, DAT-SPECT, and 123I-MIBG-SPECT imaging provides valuable information in the clinical diagnosis of multiple system atrophy (MSA). These imaging approaches offer insights into structural and metabolic alterations specific to MSA, aiding in the differentiation from other parkinsonian disorders. However, the role of dopaminergic imaging techniques in MSA diagnosis and the controversies surrounding 123I-MIBG-SPECT imaging warrant further investigation and discussion in future studies.

## Current clinical tests to aid clinical diagnosis

In addition to imaging, clinical testing such as cardiovascular, autonomic and urogenital testing are useful in the diagnosis of MSA. MSA shares the symptoms of autonomic dysfunction cerebellar ataxia, and parkinsonism with numerous other genetic diseases, such as PD and PSP, which makes diagnosis a challenge (11). A hallmark sign of MSA is neurogenic orthostatic hypotension (OH), defined as a sustained drop in systolic blood pressure of at least 30 mmHg within 3 min of standing up or head-up tilt to at least 60° (11, 18). However, OH can also occur in DLB and PD, and even when present, it may not cause noticeable symptoms unless specifically looked for. Beyond the bedside, formal autonomic testing can distinguish whether OH is neurogenic as well as detect signs of sudomotor or cardiovagal failure and assess the distribution and overall severity of autonomic failure (37).

Cerebellar ataxia, commonly a broad based gait, is seen in 36–64% of MSA patients, with a close to 100% gait ataxia seen in patients with MSA-C. Other signs of cerebellar symptoms include limb ataxia (87%–94%), postural tremor (45%) (38). Ocular symptoms are another manifestation of cerebellar abnormalities, such as positional downbeat nystagmus and saccadic hypermetria present in 23% of patients with MSA (38). The phalanx sign, tested with nose-to-finger repetitions to indicate limb dysmetria, is a bedside tool used to evaluate cerebellar dysfunction and is helpful in distinguishing MSA from other neuropathologies (38).

Parkinsonism is an umbrella term used to denotate any neuropathology that causes bradykinesia, stiffness, and tremor. In MSA, parkinsonism is symmetrical, with early postural instability characteristic of their falls. It has rapid progression in MSA with as little as 3 years of time from onset with 33% of patients requiring walking aids (38). In addition, dyskinesia in MSA is focal, and dystonia affecting cervical or distal limbs, whereas PD has generalized choreatic limb movements. The parkinsonism for MSA is also defined by poor response to Levodopa-Carbidopa treatment with studies showing 74% of patients reporting poor response to treatment (34). These features help distinguish between characteristic MSA traits and other neurodegenerative disorders.

Another characteristic symptom that aids in MSA diagnosis is urogenital dysfunction. MSA frequently damages Onuf’s nucleus, which supplies nerves to the external anal and urethral sphincters, leading to urinary frequency, urgency, and incontinence (3). Neurogenic lower urinary tract dysfunction can occur quite early in MSA and is a prognostic marker for shortened survival (22, 39). In contrast to the more common problem of an overactive bladder, patients with MSA frequently have decreased detrusor contractility (39). A postvoiding bladder scan by ultrasound is useful for screening for urinary retention, and urodynamic studies may be indicated for further evaluation and to distinguish a hypotonic bladder from urinary outlet obstruction (3, 7, 11).

Polysomnography can also be useful to detect the loss of atonia that occurs in rapid eye movement (REM) behavior disorder during sleep (3). While not mentioned within the MDS criteria, this marker can help as supporting evidence for the diagnosis of MSA. Some patients with MSA may exhibit violent motor activity during REM sleep, as reported by their bedpartners (7). This study shows REM sleep behavior disorder is present in 88% of patients with MSA, with more than half reporting these symptoms before motor deficits set in Palma et al. (40). However, this symptom is nonspecific and can also be seen in other pathologies, such as PD and in response to certain medications (41). While the clinical tests outlined above can help physicians diagnose MSA, there is currently no definitive test to diagnose MSA during life. Diagnosis relies on a combination of findings from the neurological examination, imaging studies, and clinical testing (3, 7). However, clinical diagnosis is not always accurate, highlighting the urgent need for specific biomarkers to enable accurate and early diagnosis and effective treatment (3, 7, 11).

## Structural and molecular diversity of α-synuclein between MSA and PD

The progression and severity of MSA and PD are distinct, with MSA progressing more rapidly and having a shorter life expectancy (32). MSA is specifically compared to PD as it is most often misdiagnosed as PD, due to the similarity in symptoms (9). Thus, identifying a biomarker difference that can be tested to definitively diagnose between the two can alleviate the most common misdiagnosis. The difference in disease progression may be explained by differences in the molecular structure of α-synuclein strains. Studies have shown that recombinant monomers of α-synuclein can aggregate into different conformations, and that mutations of the SNCA gene, such as A53T, A30P, E46K, G51D, and H50Q, are associated with familial PD (42, 43). In contrast, the specific substitution of G51D and A53D is associated with an MSA-like phenotype (44). These observations are consistent with other biochemical findings that show altered structural and seeding properties of α-synuclein fibrils, lipid binding, and nuclear localization (45). Additionally, amplification and analysis of α-synuclein aggregates from brain samples of MSA and PD patients reveal differences in the structural and seeding properties of the protein aggregates (42, 43, 46). The greater structural diversity of α-synuclein fibrils in PD samples, compared to MSA samples, suggests that the gradual disease course of PD allows for greater diversity in protein aggregates (46). Furthermore, the presence of different α-synuclein fibrils in each disease implies that the propagation of α-synuclein may provoke different mechanisms of neurodegeneration leading to different clinical phenotypes (33, 36).

To better understand the differences between MSA and PD, future studies are needed to identify and differentiate α-synuclein strains *in vivo* and to expand upon limited knowledge of α-synuclein aggregation in the brain (42). Recent studies are leveraging this knowledge of differences in structural and aggregation properties to develop specific aggregation-based amplification approaches targeting MSA. For instance, one study found that the MSA signal intensified more than PD when aggregation was performed in a buffered solution (40 mM PB, pH 8, 350 mM Na Citrate) (47). By studying the conformational structural diversity of α-synuclein under varying conditions, it may be possible to differentiate and diagnose neurological conditions related to α-synuclein pathology.

## Emerging biomarkers for MSA diagnosis

The diagnosis of multiple system atrophy (MSA) presents a clinical challenge, as definitive diagnosis is only possible via autopsy and histopathology challenging inclusion of accurate patients in clinical trials (48). This poses an inherent difficulty in accurately diagnosing patients in practice and in subject selection for developing early diagnostics and interventions. There is a pressing need to discover a evidence-based biomarker to develop interventions that slow or halt the progression of the disease and improve patient care, as well as to enhance the validity of clinical trials (49, 50).

Biochemical and molecular methodologies such as ELISA, PCR, and gene expression have been used to study the aggregation of α-synuclein, a promising disease-specific biomarker that aggregates in glial cells (21). ELISA is an antibody-based detection method that quantitates the concentration of a specific antigen with clinical relevance and biomarker value in MSA (51). Although a consistent and reproducible biomarker has not been found, a combination of analytics has shown potential. Neurofilament light chain (NfL), a structural element of neuronal cells released upon cellular damage, was consistently elevated in MSA compared to controls or PD, indicating neuronal degradation (17); Additionally, an ELISA antibody for Ser129 phosphorylated α-synuclein showed success in detection and more recent advances include development and testing of phospho-specific antibody for variety of human specimens (51, 52). Transgenic mouse models (M83) based on the α-synuclein seeding mechanism demonstrated an increased accumulation of insoluble α-synuclein within the involving prion-like spread (51).

Other earlier biomarker approaches used quantitative PCR, which measures the RNA expression of the *SNCA* gene responsible for coding the α-synuclein protein which have been observed to cause parkinsonism disorders (53). However, this study showed no difference in mRNA expression for the *SNCA* gene in MSA compared to non-MSA samples (42). Later studies using RNA sequencing approaches to evaluate whether increased *SNCA* expression was related to the diagnosis of MSA were also inconclusive (1, 54). Genomic multiplication of *SNCA* suggested a pathogenic hypothesis such that MSA was related to over-expression of the α-synuclein protein (53), and DNA sequencing of the exons of the *SNCA* gene showed that MSA was not driven by rare coding mutations (53). As the exons would have shown the sequences that were being converted to proteins, thereby providing insight to the protein structure, and folding, as MSA is thought to be due to the misfolding of the α-synuclein protein. However, Genome-wide association studies (GWAS) studies involving MSA lacked desired sample sizes and were inconclusive as no significant genetic variants were identified specific to MSA incidence (1, 55).

Seeding assays for α-synuclein have recently emerged as a novel approach to identify molecular biomarkers. Two major methods currently employed to study aggregated α-synuclein include protein misfolding cyclic amplification (PMCA) and real-time quaking-induced conversion (RT-QuIC) (49, 56). These assays utilize amplification of a small amount of seeding competent, misfolded α-synuclein from human samples and biofluids. As α-synuclein amplification is the main hypothesis driving the pathophysiology of MSA, PMCA and RT-QuIC provide a method of quantifying and comparing the amount of synuclein to extrapolate a relationship with symptoms seen in disease. PMCA is one method of amplification of protein, which in the case of MSA is α-synuclein, that allows for detection by enhancing the small amount present in biofluids (48). RT-QuIC works via a pathogenic seed from the patient, in the case of MSA with α-synuclein, and intermittent shaking is utilized to encourage an interaction with the seed and the substrate which allow for measurement of the conversion from monomers to polymers (49). The thermodynamics of this assay occurs either through shaking in the case of RT-QuIC assays or sonication in PMCA assays. The reactions are monitored in a controlled environment in real time using a fluorophore such as Thioflavin T that emits a signal when bound to fibrillar structures present in seeded and misfolded α-synuclein. This allows for aggregated protein to break up and continue to propagate more aggregation, thereby allowing us to measure the amplification of misfolded protein (49, 57). The versatility of these methods has allowed for the detection of abnormal conformations of misfolded protein in a variety of human tissues and biofluids, including cerebrospinal fluid, olfactory mucosa, saliva, and blood (57–61). Furthermore, RT-QuIC has proven successful in easily accessible peripheral tissues such as skin (60, 62), submandibular glands (63) as well as in colon biopsies. Using similar seeding-based approaches these discoveries have been independently replicated by other laboratories and clinical settings (59, 61, 62, 64, 65). The consistency of detection and versatility of RT-QuIC for various tissues and biofluids has established this method as a gold standard in the study of α-synuclein protein aggregation in human specimens (Table 1).

**Table 1:** Molecular biomarkers using biofluid for multiple system atrophy (MSA) patients.

Biomarker	Sample type and size	Autopsy confirmed	Major observations of the study	References
𝛂-**synuclein ELISA and seeding-based approaches**				
Antibodies to 𝛂-synuclein	*Plasma*	N	- IgG autoantibodies are absent in MSA, decreasing the ability to clear pathologic α-synuclein.	(66)
	MSA (*n* = 34)			
	PD (*n* = 43)		- Anti α-synuclein IgM decreased in MSA and PD. In addition, MSA had reduced IgG and IgM compared to PD and control. This suggests they have distinct immune pattern.	
	Control (*n* = 59)			
𝛂-synuclein seeding	*CSF*	Y	- Three predictors for the diagnostic probability of a diagnosis of synucleinopathies—age, CSF tau, and CSF α-synuclein with *p* < 0.0001.	(67)
	AD (*n* = 62)			
	Control (*n* = 76)			
	DLB (*n* = 55)		- CSF α-synuclein concentration was lower in PD, dementia with LB, and MSA than in other neurological diseases.	
	PD (*n* = 51)			
	MSA (*n* = 29)			
𝛂-synuclein seeding and NfL	*CSF*	N	- NfL was elevated in MSA compared to control, PD, and DLB *p* < 0.001. No difference between MSA-P and MSA-C.	(68)
	Discovery cohort:		- Using NfL > 1,400 pg./mL and ThT fluorescence, MSA can be differentiated with 100% sensitivity and 83% specificity.	
	MSA patients (*n* = 24)			
	Control (*n* = 14)			
	Prospectively enrolled cohort:			
	MSA (*n* = 38),		- NFL and αSyn seeding in CSF differentiate MSA from healthy controls and Lewy body synucleinopathies.	
	PD (*n* = 16),			
	Control (*n* = 15)			
NfL and NG2 measurement	*CSF*	N	- CSF levels of NfL and NG2 are higher in MSA than in control. Correlation between α-synuclein and NfL (r = 0.423).	(69)
	MSA (*n* = 50)			
	Control (*n* = 20)		- Diagnosis via CSF NfL for MSA was the sensitivity of 100% and specificity of 100%. No significant difference was found between MSA-P and MSA-C regarding NfL measurement.	
𝛂-synuclein levels in Extracellular vesicles/-exosomes	*Plasma*	N	- Oligodendrocyte-derived exosomes (ODE) did not correlate with disease duration or severity in MSA-C but did correlate in MSA-P.	(70)
	PD (*n* = 15)			
	MSA (*n* = 15)		- Plasma levels of Neuronal derived exosomes (NDE) were higher in PD than in MSA or control.	
	PSP (*n* = 7)		- Plasma levels of Oligodendrocytes derived exosomes and ODE: NDE ratio significantly correlated with UPDRS part III scores for MSA-P.	
	Control (*n* = 15)			
Exosomes derived from blood	*Serum or plasma*	Y	- Analysis of samples showed decreased exosomes in control than in PD or MSA. α-synuclein is increased in MSA and PD compared to control.	(21)
	Postmortem cohort			
	PD (*n* = 49)			
	MSA (*n* = 12)		- Analysis of oligodendrocyte synuclein showed more in MSA than PD with a sensitivity of 90% and specificity of 88.2%.	
	*Validation cohort*			
	PD (*n* = 50)			
	MSA (*n* = 50)		- The analysis of α-synuclein concentration and exosome concentration showed separation of PD from MSA with an AUC of 0.902, corresponding to 89.8% sensitivity and 86.0% specificity.	
	Control (*n* = 50)			
**𝛂-synuclein seeding (RT-QuIC) only based approaches**				
𝛂-synuclein seeding	*Saliva*	N	- Electron microscopy of α-synuclein in the sample showed negative staining, indicating they can perform seeding activity using saliva.	(71)
	MSA (*n* = 18)		- PD and MSA α-synuclein fibrils showed no significant difference in diameter (*p* > 0.05) in α-synuclein fibrils.	
	PD (*n* = 75)			
	HC (*n* = 36)		- RT-QuIC performed on saliva samples of patients detected MSA from control with 61.1% sensitivity and PD from control with 76% sensitivity and 94% specificity.	
𝛂-synuclein seeding	*Olfactory mucosa*	N	- 19/29 olfactory mucosa (OM) samples showed α-synuclein seeding activity showing OM may be used instead of CSF for potential neurodegenerative disease diagnosis.	(58)
	PD (*n* = 18)			
	MSA (*n* = 11)		- Seeding showed different structures of α-synuclein aggregation present with different synuclein strains. RT-QuIC can be utilized in future studies to determine the sensitivity and specificity of OM samples.	
	CBD (*n* = 6)			
	PSP (*n* = 12)			
𝛂-synuclein seeding	*Olfactory mucosa*	N	- 18/20 patients with MSA-P showed seeding activity via OM samples with a sensitivity of 90%. MSA-C showed no seeding activity.	(57)
	PD (*n* = 13)		- Seeding activity of synuclein correlated with motor symptoms such as rigidity and instability.	
	MSA-P (*n* = 20)			
	MSA-C (*n* = 10)		- RT-QuIC of OM samples for α-synuclein can detect seeding from PD, and MSA-P but not MSA-C.	
	HS (*n* = 11)			
𝛂-synuclein seeding	*CSF*	N	- Patients with MSA showed higher levels of plasma NfL than patients with PD.	(72)
	PD (*n* = 153)			
	MSA (*n* = 80)			
	PSP (*n* = 58)		- RT-QuIC detected synuclein in 2.5% of MSA. PD showed 91.4% detection of synuclein.	
	DLB (*n* = 64)			
	REM sleep disorder (*n* = 19)			
	Isolated autonomic failure (*n* = 30)		- Analysis and ROC curves showed high accuracy via CSF samples with 95.7% sensitivity and 100% specificity in differentiating PD and MSA.	
𝛂-synuclein seeding and NfL	*Skin and serum*	Y	- α-synuclein was also analyzed and was detected in 80% MSA, 77% PD, 14% PSP, and 15% HC.	(61)
	MSA (*n* = 10)		- Serum NfL with a threshold of 30 pg./mL along with RT-QuIC showed 100% sensitivity and 100% specificity in differentiating MSA from PD.	
	PD (*n* = 13)		- 100% sensitivity and 93% specificity in distinguishing MSA from PD, PSP, and Control.	
	PSP (*n* = 7)			
	HC (*n* = 20)			
𝛂-synuclein seeding	*CSF*	N	- Findings showed differences between intensity of fluorescence and aggregation speed between MSA and PD, with PD aggregating slower but with higher fluorescence via amyloid dye.	(73)
	PD (*n* = 94)		- Aggregates of MSA samples have more beta sheets of α-synuclein than aggregates of PD samples.	
	MSA (*n* = 75)			
	Control (*n* = 56)		- Sensitivity of PD is 93.6% with a specificity of 100% while MSA showed a sensitivity of 84.6% with 100% specificity.	

## Current developments in molecular biomarkers for MSA

Recent studies have also focused on measuring α-synuclein in extracellular vesicles (EVs) as a potential non-invasive biomarker for diagnosing MSA. EVs are small membrane-bound particles released by all cells, including neurons, and contain proteins, RNA, and DNA that reflect the functions and processes of the brain (74). EVs have been shown to carry various biomarkers, including α-synuclein, which can indirectly allow for the measurement of brain-derived α-synuclein (20, 61). While detecting synuclein aggregation in the brain is challenging during a patient’s lifetime, the exosomes released into biofluids such as CSF and blood can be isolated using brain cell surface markers (20). In one study, the analysis of exosomes extracted from a blood sample was able to differentiate between MSA and PD with 90% accuracy (21). Moreover, recent developments have explored alternative sources for obtaining biomarkers for MSA diagnosis with minimal invasiveness. α-synuclein seeding from tissues with less invasive sites such as skin, olfactory mucosa, gastrointestinal mucosa, and blood have shown promising results and may serve as potential screening methods (49, 75). Further research is being conducted to optimize and validate the use of these biomarkers, with the aim of establishing a non-invasive method for diagnosing MSA in routine clinical practice. These efforts also include exploring the correlation between the levels of biomarkers and MSA during the early stages of disease progression, which may aid in the early detection and treatment of MSA. As shown in Table 2, several clinical trials are currently underway to investigate the potential of various molecular biomarkers in diagnosing MSA, highlighting the growing interest in this field and the need for further research to establish accurate and reliable diagnostic tools.

**Table 2:** Clinical and molecular biomarker outcomes currently being implemented in clinical trials.

Biomarker	Outcome measure and number of samples tested	Autopsy confirmed	Findings and Statistics	Implemented in clinical trials?	References
α-synuclein in plasma derived EVs	blood test, (*n* = 10)	Y	90% accuracy in differentiating MSA	N	(21)
α-synuclein	CSF, (*n* = 48)	N	Measure total free vs. oligomeric synuclein in CSF	Y	NCT01485549
α-synuclein aggregates in CSF by qRT-QuIC	CSF samples, (*n*=)	Y	RT-QuIC can be used to detect synuclein aggregation	N	(76)
Plasma exosomal IRS-1pS312	Blood-based, (*n* = 124)	N	The ongoing clinical trial, pending results	Y	NCT04250493
Neurofilament light chain (NfL) levels	CSF samples, (*n* = 50)	N	The ongoing clinical trial, pending results	Y	NCT04450992
Serum miRNAs miR-30c-5p signature	Serum, (*n* = 155)	Y	PCR is used to detect miRNA in serum	N	(77)
Gait analysis	Clinical testing (*n* = 180)	N	The ongoing clinical trial, pending results	Y	(78) NCT04608604

## The current state of the art for diagnostic criteria and inclusion in clinical trials

Currently, there is no cure or disease-modifying treatment for MSA, and clinical trials have not been successful in identifying effective treatments due to the lack of confidence in the clinical diagnosis. This is mainly due to moderate rates of misdiagnosis while patients are alive, which can lead to the erroneous inclusion of misdiagnosed subjects in clinical trials for MSA studies, potentially leading to inaccurate results. Hence, the availability of a specific molecular biomarker that could provide an accurate diagnosis is vital to the accuracy and validity of clinical trials to study MSA (9, 14, 19).

**Figure 2:**
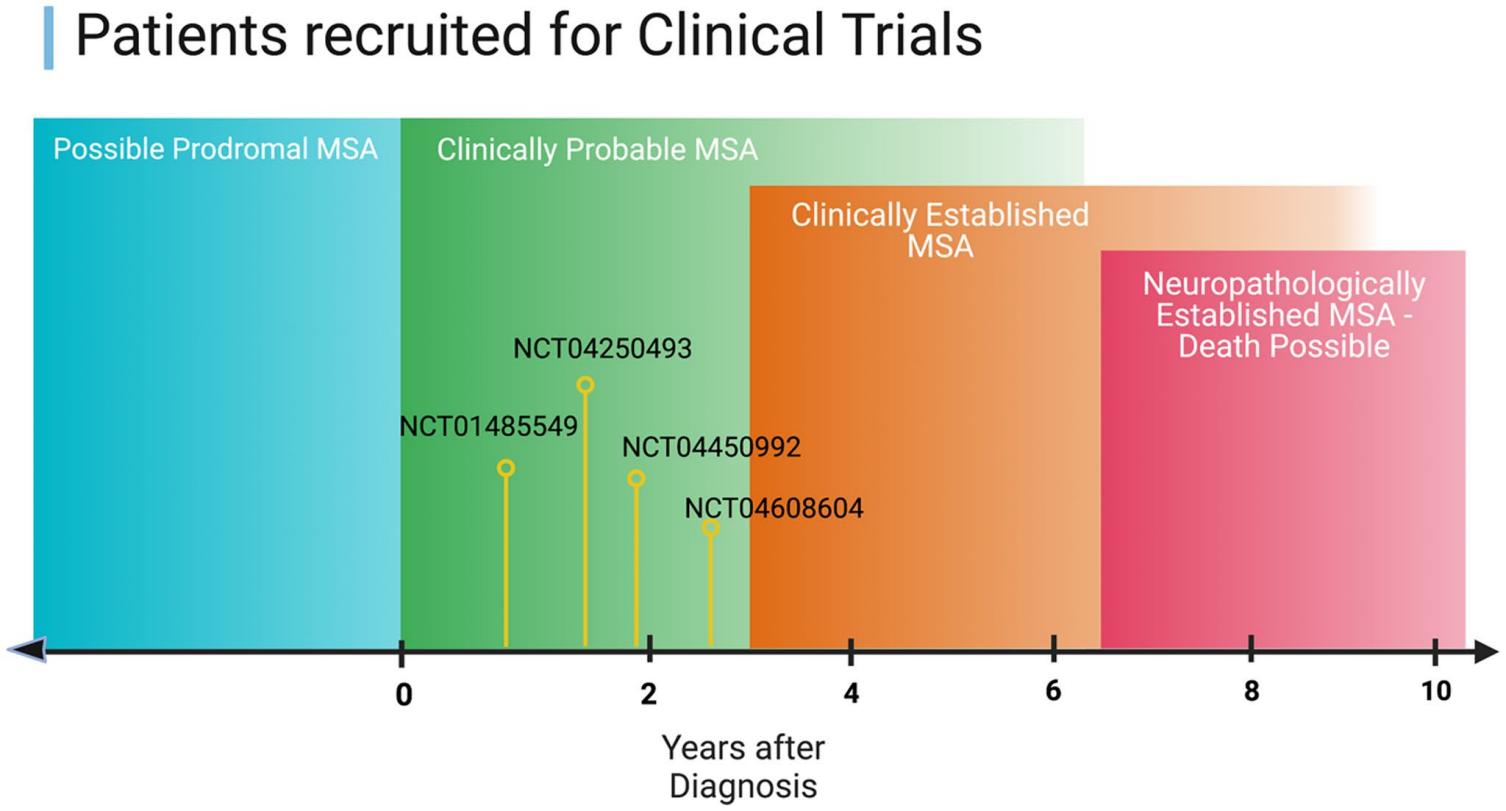
Illustration of the current progress on the therapeutics investigated in clinical trials in the context of MSA disease stage.

The method that most clinical trials use for their inclusion criterion is based on the clinical biomarkers outlined by Gilman [ClinicalTrials.gov] Reference: NCT03952806, UMIN-CTR. For inclusion, the subject is required to have probable or possible MSA as defined in Figure 2. As such, most clinical trials rely on clinical biomarkers, which are still prone to error and bias. Other trials utilize MRI and require findings consistent with MSA as an inclusion criterion, although other disorders can show similar findings, leading to inaccurate inclusion or exclusion of participants in the trial [Reference: NCT04184063].

Current standards for MSA diagnosis and inclusion criteria involve the onset of autonomic MSA symptoms up to 5 years before a screening visit [ClinicalTrials.gov] Reference: NCT05104476. Some trials utilize the Unified Multiple System Atrophy Rating Scale (UMSARS) and the Montreal Cognitive Assessment (MOCA) for inclusion criteria, but these still contain a degree of bias. The UMSAR was developed in the early 2000s as a method of disease specific rating device utilized to measure things such as functional disability, bloop pressure and heart rate, and motor impairment, with a higher score dictating increased disability to quantitatively compare patients (79). The MOCA is another assessment tool for cognitive impairment consisting of a 30 point test with a quantitative score. A score of over 26 indicates normal cognitive function (80). One study found that there was no significant difference in cognitive ability when comparing MSA patients with controls via MOCA. However, the type of cognitive impairment seen in MSA patients with MOCA is specifically within the domains of visuo-spatial and executive functions whereas the control mainly had decline regarding language and abstraction (81). UMSARS, which is a scaled measure that correlates with disease progression of MSA, with numerical cutoffs for inclusion [Reference: NCT05167721]. The Montreal Cognitive Assessment (MOCA) with a score of >26 is also utilized as a measure of cognitive function for inclusion criteria [Reference: NCT05167721]. The UMSARS Part one score of less than 16 as well as the Montreal Cognitive Assessment with a score greater than 22 are assessed at the screening visit [Reference: NCT05104476]. Reliance on clinical biomarkers leads to potential errors in candidate selection for clinical trials, which could be greatly improved by the development of a specific molecular biomarker. Therefore, early diagnostic biomarkers could play a crucial role in better improving the early diagnosis of MSA and enabling early interventions.

## Conclusion

Multiple system atrophy (MSA) is a devastating disorder with limited treatment options and no cure. MSA is a rapidly progressing neurodegenerative disorder that is characterized by a decrease in motor ability leading to death 5–7 years after initial diagnosis (82). The current clinical biomarkers used for diagnosis lack specificity and frequently result in misdiagnosis or delayed diagnosis. In addition, clinical testing to diagnose MSA, such as MRI and CSF analysis, can be expensive and intrusive with low specificity rates (83). The most recent technology to detect early MSA includes *in vivo* PET imaging of α-synuclein depositions, although detection in human models has not been proven and the procedure is expensive (31).

Recent technological advancements have led to the development of new molecular biomarkers for the diagnosis of MSA. One promising approach is the detection of α-synuclein in biological fluids such as blood, plasma, and urine. Other studies have investigated the use of NfL and glial fibrillary acidic protein (GFAP) as biomarkers for MSA diagnosis, although further validation studies are needed (61, 72, 84). The development of accurate molecular biomarkers for MSA diagnosis is essential, as it could revolutionize the diagnosis and treatment of MSA, allowing for more timely patient care. In addition, accurate diagnosis at an early stage of the disease would facilitate disease-modifying interventions, once available, to be more effective.

Currently, most studies are feasible only after clinical signs of disease are apparent, as illustrated in Figure 2 of this manuscript. Therefore, targeting prodromal stages for clinical trials could be crucial as therapeutics are thought to be much more effective in the earlier stages of the disease. Moreover, the search for a molecular biomarker that can be used in clinical trials could improve the development of new therapies and provide better insights into the mechanisms of MSA.

In conclusion, the development of molecular biomarkers for the diagnosis of MSA holds promise toward providing an effective, noninvasive, and scalable method of diagnosing MSA and distinguishing it from look-alike disorders. This could also remove the burden of ineffective treatment due to misdiagnosis or delayed treatment due to late diagnosis. Further research to find a molecular biomarker to diagnose MSA is vital to better understand this condition and improve treatment methods leading to better patient outcomes.

## Author contributions

NK and MG contributed equally to the conception and design of this review article. MG conducted the literature review and drafted the initial manuscript, NK, OR, and WC provided critical revisions and feedback, and helped to finalize the manuscript. All authors contributed to the article and approved the submitted version.
